# Retraction Note To: Pluronic-based nano-self-assemblies of bacitracin A with a new mechanism of action for an efficient in vivo therapeutic effect against bacterial peritonitis

**DOI:** 10.1186/s12951-022-01611-6

**Published:** 2022-09-13

**Authors:** Wei Hong, Lipeng Liu, Yining Zhao, Yinghui Liu, Dexian Zhang, Mingchun Liu

**Affiliations:** Key Laboratory of Zoonosis of Liaoning Province, College of Animal Science and Veterinary Medicine, Shenyang Agricultural University, Dongling Road 120, Shenyang, 110866 Liaoning People’s Republic of China

## Retraction to: J Nanobiotechnol (2018) 16:66 https://doi.org/10.1186/s12951-018-0397-3

The Editors-in-Chief have retracted this article due to concerns with Figure 1. After publication, the authors requested replacement of Figure 1 with a new version. In the new version, the sizes of the nanoparticles appear to be much smaller than the reported sizes in Table 1, raising concerns about the reliability of the reported data. Further, it was noted that there was overlap in the images in the published version of Figure 1A and 1C. In light of these concerns, the Editors-in-Chief no longer have confidence in the integrity of the data in this article.

Mingchun Liu agrees with the retraction. Wei Hong does not agree with the retraction. The other authors did not respond to any correspondence from the editor or publisher about the wording of this retraction notice.

